# Supplementary figures and images for: Altered myocardial lipid regulation in junctophilin-2–associated familial cardiomyopathies
Source: Life Sci Alliance. 2024 Mar 4;7(5):e202302330. doi: 10.26508/lsa.202302330 (PMC10912815; doi:10.26508/lsa.202302330)

**Fig 1A-B**

**A399S**

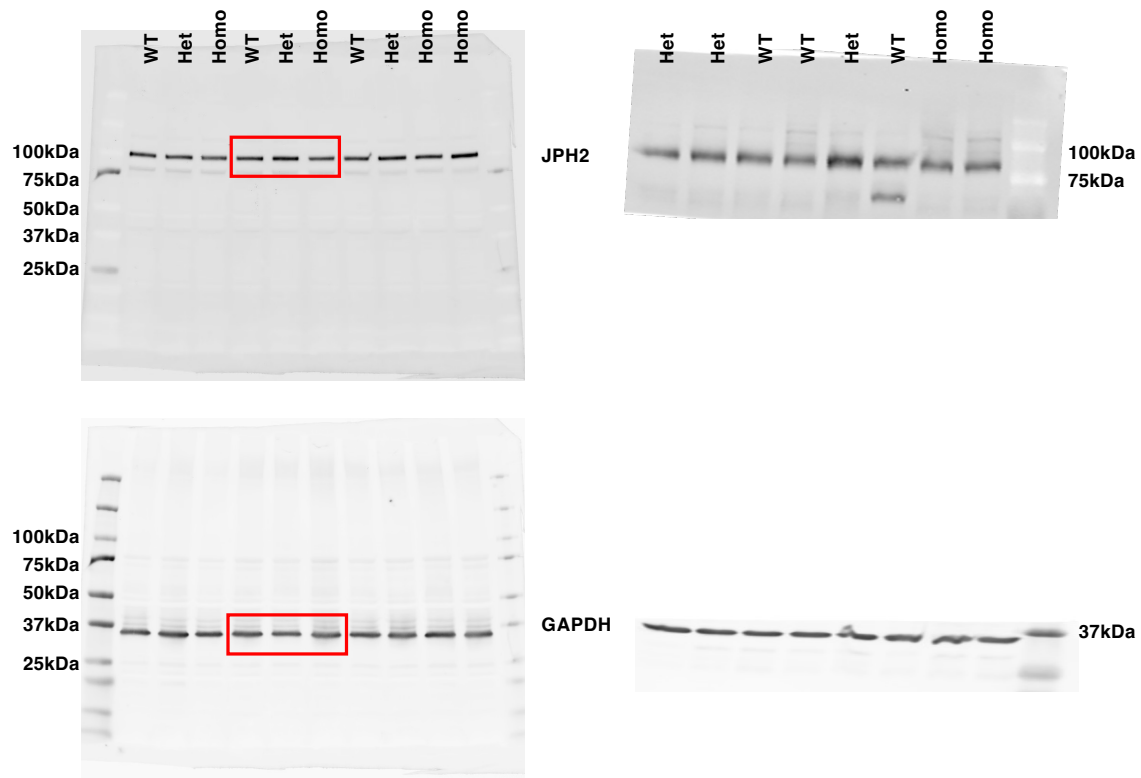

**Fig 1C-D**

**E641\***

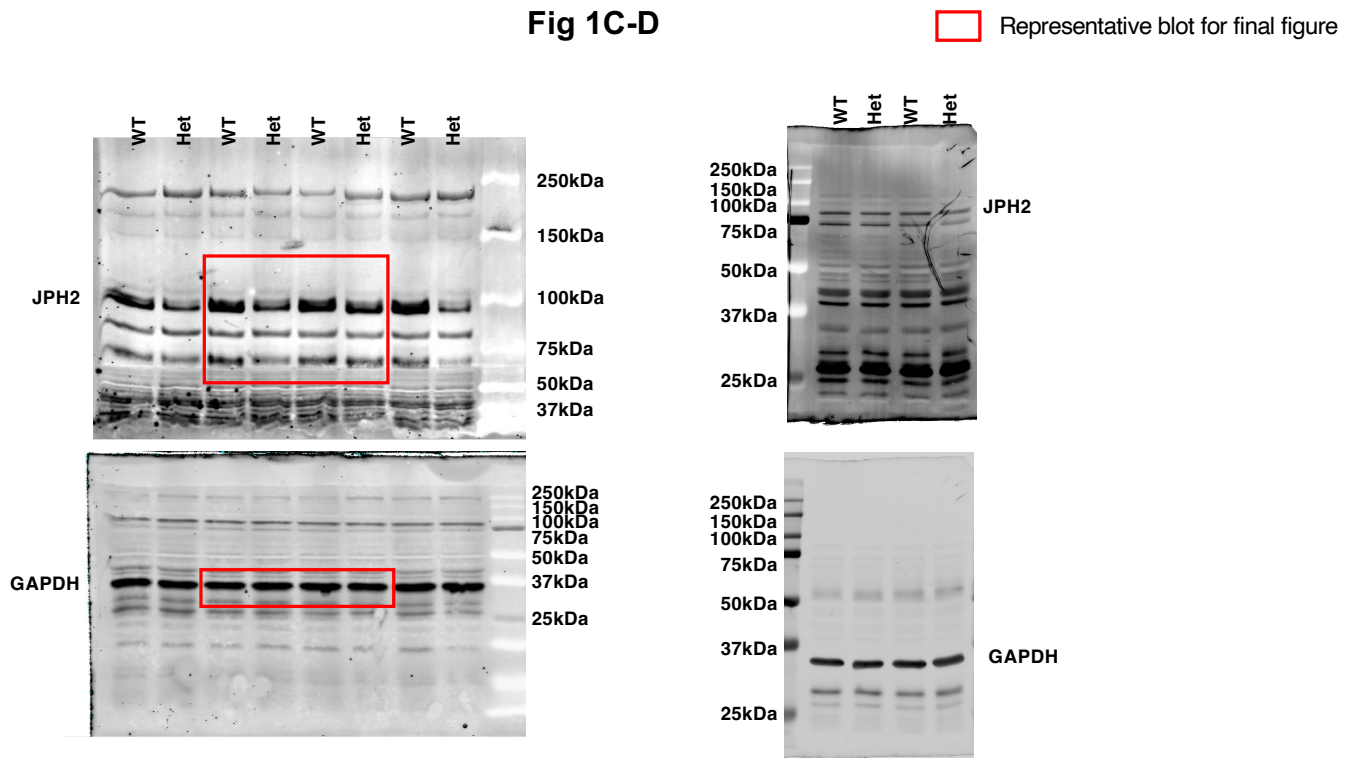

Supplement: Supplementary file 1 [file LSA-2023-02330_SdataF1.pdf]
