# Supplementary material for: Altered myocardial lipid regulation in junctophilin-2–associated familial cardiomyopathies
Source: Life Sci Alliance. 2024 Mar 4;7(5):e202302330. doi: 10.26508/lsa.202302330 (PMC10912815; doi:10.26508/lsa.202302330)
Supplement: Supplementary file 2 [file LSA-2023-02330_TableS1.docx]

**Supplemental Table 1. Echo parameters from A399S and E641* knock-in mice.**

|  | **WT**  **(N=6)** | **A399S-Het (N=6)** | **A399S-Homo (N=6)** | **P value**  **(Het vs. WT)** | **P value**  **(Homo vs. WT)** |
| --- | --- | --- | --- | --- | --- |
| **IVS;d (mm)** | 1.00 ± 0.01 | 1.14 ± 0.04 | 1.25 ± 0.04 | **0.007** | **<0.001** |
| **LVEDD (mm)** | 3.59 ± 0.09 | 3.52 ± 0.08 | 3.59 ± 0.09 | 0.580 | 0.989 |
| **LVESD (mm)** | 2.27 ± 0.1 | 2.25 ± 0.1 | 2.23 ± 0.07 | 0.923 | 0.738 |
| **LVEF (%)** | 67.75 ± 1.64 | 66.68 ± 2.04 | 69.07 ± 1.29 | 0.690 | 0.541 |
| **LVFS (%)** | 37.01 ± 1.2 | 36.23 ± 1.52 | 38.05 ± 1 | 0.693 | 0.523 |
| **LVEDV (uL)** | 54.45 ± 3.23 | 51.93 ± 2.73 | 54.41 ± 3.16 | 0.565 | 0.994 |
| **LVESV (uL)** | 17.81 ± 1.92 | 17.6 ± 1.87 | 16.91 ± 1.28 | 0.940 | 0.706 |
| **SV (uL)** | 36.64 ± 1.4 | 34.33 ± 1.15 | 37.5 ± 2.13 | 0.230 | 0.743 |
| **CO (mL/min)** | 19.87 ± 0.62 | 17.99 ± 0.5 | 21.19 ± 1.32 | 0.040 | 0.389 |
| **LVAW;d (mm)** | 0.81 ± 0.04 | 0.79 ± 0.03 | 0.8 ± 0.03 | 0.777 | 0.814 |
| **LVAW;s (mm)** | 1.14 ± 0.05 | 1.03 ± 0.03 | 1.08 ± 0.03 | 0.073 | 0.307 |
| **LVPW;d (mm)** | 0.83 ± 0.02 | 0.79 ± 0.02 | 0.87 ± 0.02 | 0.117 | 0.157 |
| **LVPW;s (mm)** | 1.08 ± 0.02 | 1.04 ± 0.03 | 1.09 ± 0.04 | 0.378 | 0.769 |

|  | **WT (N=8)** | **E641* (N=9)** | **P value** |
| --- | --- | --- | --- |
| **LVEDD (mm)** | 3.47 ± 0.15 | 3.69 ± 0.14 | 0.146 |
| **LVESD (mm)** | 2.20 ± 0.16 | 2.78 ± 0.10 | **0.008** |
| **LVEF (%)** | 67.5 ± 3.38 | 49.6 ± 1.23 | **<0.001** |
| **LVFS (%)** | 37.1 ± 2.62 | 24.6 ± 0.77 | **<0.001** |
| **LVEDV (uL)** | 50.8 ± 4.67 | 58.7 ± 5.01 | 0.272 |
| **LVESV (uL)** | 17.3 ± 2.77 | 29.6 ± 2.75 | **0.007** |
| **SV (uL))** | 35.3 ± 2.34 | 31.0 ± 2.45 | 0.226 |
| **CO (mL/min)** | 17.3 ± 1.26 | 15.7 ± 1.24 | 0.406 |
| **LVAW;d (mm)** | 0.80 ± 0.04 | 0.87 ± 0.04 | 0.263 |
| **LVAW;s (mm)** | 1.19 ± 0.07 | 1.23 ± 0.07 | 0.700 |
| **LVPW;d (mm)** | 0.81 ± 0.04 | 0.85 ± 0.04 | 0.499 |
| **LVPW;s (mm)** | 1.18 ± 0.04 | 1.07 ± 0.05 | 0.101 |
